# Control of neglected tropical diseases needs a long-term commitment

**DOI:** 10.1186/1741-7015-8-67

**Published:** 2010-10-29

**Authors:** Yaobi Zhang, Chad MacArthur, Likezo Mubila, Shawn Baker

**Affiliations:** 1 Helen Keller International, Regional Office for Africa, Dakar, Senegal; 2 Helen Keller International, Headquarters, New York, the USA; 3 World Health Organization, Regional Office for Africa, Harare, Zimbabwe

## Abstract

**Background:**

Neglected tropical diseases are widespread, particularly in sub-Saharan Africa, affecting over 2 billion individuals. Control of these diseases has gathered pace in recent years, with increased levels of funding from a number of governmental or non-governmental donors. Focus has currently been on five major 'tool-ready' neglected tropical diseases (lymphatic filariasis, onchocerciasis, schistosomiasis, soil-transmitted helminthiasis and trachoma), using a package of integrated drug delivery according to the World Health Organization guidelines for preventive chemotherapy.

**Discussion:**

Success in controlling these neglected tropical diseases has been achieved in a number of countries in recent history. Experience from these successes suggests that long-term sustainable control of these diseases requires: (1) a long-term commitment from a wider range of donors and from governments of endemic countries; (2) close partnerships of donors, World Health Organization, pharmaceutical industries, governments of endemic countries, communities, and non-governmental developmental organisations; (3) concerted action from more donor countries to provide the necessary funds, and from the endemic countries to work together to prevent cross-border disease transmission; (4) comprehensive control measures for certain diseases; and (5) strengthened primary healthcare systems as platforms for the national control programmes and capacity building through implementation of the programmes.

**Conclusions:**

The current level of funding for the control of neglected tropical diseases has never been seen before, but it is still not enough to scale up to the 2 billion people in all endemic countries. While more donors are sought, the stakeholders must work in a coordinated and harmonised way to identify the priority areas and the best delivery approaches to use the current funds to the maximum effect. Case management and other necessary control measures should be supported through the current major funding streams in order to achieve the objectives of the control of these diseases. For a long-term and sustainable effort, control of neglected tropical diseases should also be integrated into national primary healthcare systems.

## Background

A group of chronic and debilitating conditions, caused by parasitic, bacterial, and viral infections, is defined as the neglected tropical diseases (NTDs) [1,2]. These NTDs are the most prevalent diseases in the poorest populations in the world, putting an estimated 2.7 billion people at risk [3]. They cause blindness (for example, onchocerciasis and trachoma) [4-7], disfigurement (for example, lymphatic filariasis, leishmaniasis, leprosy, and Buruli ulcer) [8-12], and are often life threatening at a later stage of the disease (for example, African trypanosomiasis, Chagas disease, dengue fever, and schistosomiasis) [13-17]. They are also related to various clinical complications, such as anaemia and other forms of malnutrition (for example, hookworm infections, schistosomiasis, ascariasis, and trichuriasis) [18-23]. In terms of disability-adjusted life years (DALYs), NTDs as a whole are among the top 10 leading causes of years of healthy life lost to long-term disability and premature death worldwide [1,3]. Sub-Saharan Africa bears the biggest burden of many of these NTDs, and the numbers of people afflicted by each of several of these diseases are simply striking [24], representing up to over 90% of the world's burden for some of these diseases. Moreover, these NTDs geographically overlap [1], and a significant proportion of the poorest populations often harbours more than one of these NTDs [2,25,26]. These NTDs have severe socioeconomic consequences as they cause long-term illness, disfigurement, social stigma and marginalisation, and decreased productivity [27,28]. Therefore, successful control of NTDs will have a wide range of health and socioeconomic benefits to the poorest populations. The control of NTDs has now gained momentum in recent years with growing interest from the international community and funding from governmental or non-governmental donors, however more donors are needed to provide the funds to bridge the funding gap; the existing donors need to rethink their funding policy to include other necessary control measures for certain diseases in order to achieve the objectives, and the endemic countries need to commit themselves to the NTD control effort. This paper reviews experiences in the success of controlling these NTDs from recent history, and highlights the important aspects in the control of these NTDs in order to achieve a long-term sustainable effect.

## Discussion

### Current situation in controlling the five major NTDs

Control of the NTDs currently focuses on five major 'tool-ready' NTDs, lymphatic filariasis, onchocerciasis, schistosomiasis, soil-transmitted helminthiasis (hookworm infections, ascariasis, and trichuriasis) and trachoma in an integrated control programme [29]. This is because the drugs needed for these five NTDs are robust, safe, low cost and available by donation from pharmaceutical companies or by purchasing at relatively low costs. The current control strategy is mass drug administration (MDA) using the available drugs in a coordinated approach according to World Health Organization (WHO) guidelines on preventive chemotherapy [30]. This drug-based intervention delivers available drugs either alone or in combination to prevent morbidity caused by these NTDs, or in some cases to eliminate the diseases. To date, with the funds committed to deliver the available tools to the poorest people in need from external donors, a number of countries, for example, Bangladesh, Burkina Faso, Cameroon, Democratic Republic of Congo, Burundi, Ghana, Haiti, Mali, Nepal, Niger, Rwanda, Sierra Leone, Southern Sudan, Tanzania, Togo, and Uganda, have been able to implement integrated national NTD control programmes. Although the current strategy emphasises integrated control by preventive chemotherapy, control of each of these five major NTDs has its own endpoint targets and its own comprehensive measures to achieve these targets (Table 1).

**Table 1:** Risk factors, comprehensive control measures and endpoint control targets for each of five major neglected tropical diseases (NTDs)

Disease	Major factors for human infection	Comprehensive control measures	Endpoint targets of control
Onchocerciasis	Parasites Black flies (transmitting infective larvae during blood feeding) Human activities (near black fly breeding sites)	Treatment with ivermectin Health education for self-protection Case management	Elimination

Lymphatic filariasis	Parasites Mosquitoes (transmitting infective larvae during blood feeding) Poor sanitation (mosquito breeding sites) Human behaviour (without self-protection)	Treatment with albendazole plus ivermectin or diethylcarbamazine Self-protection (bed net) Health education for behavioural change Hygiene and sanitation Case management	Elimination as a public health problem worldwide by the year 2020

Schistosomiasis	Parasites Intermediate host snails in fresh water releasing infective larvae Poor hygiene and sanitation Human behaviour (water contact and defecating/urinating in/near water)	Treatment with praziquantel Snail management Health education for behavioural change Hygiene and sanitation Clean water supply Hospitalisation of severe cases	Morbidity control

Soil-transmitted helminthiasis	Parasites Poor hygiene and sanitation Human behaviour (passing eggs to the environment in faeces, barefoot, not washing hands, and so on)	Treatment with albendazole or mebendazole Health education for behavioural change Hygiene and sanitation Clean water supply	Morbidity control

Trachoma	Bacteria Poor hygiene and sanitation Human behaviour (lack of facial washing)	SAFE strategy: Surgical case management (S) Treatment with azithromycin (A) Clean water supply for facial washing (F) Hygiene and sanitation in the environment (E) Health education for behavioural change	Elimination as a blinding disease worldwide by the year 2020

### Onchocerciasis

Onchocerciasis, or river blindness, is the second highest infectious cause of blindness in the world, and is now mainly distributed in 19 African countries, some foci in Yemen and parts of the Americas. There are 37 million people with onchocerciasis and 99% of the cases are in Africa, with more than 102 million people at risk of the disease in Africa alone [31]. The control strategy is through mass administration of ivermectin (Mectizan) donated by Merck & Co., Inc. through its Mectizan Donation Program http://www.mectizan.org.

In the Americas, the Onchocerciasis Elimination Program for the Americas (OEPA) was launched in 1992 to act as a technical and coordinating body for a multinational, multiagency coalition that facilitated the establishment of programmes for the semiannual mass administration of ivermectin in the six countries with onchocerciasis [32]. Significant progress has already been made in all six countries, with no new cases of onchocercal blindness being reported in the region, and it is anticipated that onchocerciasis could be eliminated from most of the remaining foci in the Americas by 2012 [32].

In Africa, the most successful control programme has been the Onchocerciais Control Program (OCP) which virtually eliminated river blindness from 11 countries first by vector control through spraying insecticides and subsequently through delivery of ivermectin [33]. In 1995, onchocerciasis control was expanded to include areas outside the original OCP zone with a second programme, the African Program of Onchocerciasis Control (APOC). It adopted a strategy of community-directed treatment with ivermectin (CDTI) annually [34], delivering over 50 million treatments per year for onchocerciasis in 19 participating countries [31]. Through this strategy, the prevalence of onchocerciasis in these countries has been reduced from a precontrol level of 46.5% in 1995 to 28.5% in 2008 [31]. In some countries, for example, Senegal and Mali, longitudinal studies in three foci showed that after 15-17 years of annual or biannual CDTI, prevalence of onchocerciasis was reduced to < 1% [35]. This has ignited a hope of eliminating onchocerciasis from Africa.

### Lymphatic filariasis

Lymphatic filariasis (LF), or elephantiasis, is widely distributed throughout the tropical and subtropical areas of Asia, Africa, the Western Pacific and some parts of the Americas [36,37]. Globally there are 120 million people infected with the parasite, causing tremendous morbidity, with 1.3 billion people at the risk of infection around the world [3,36]. Successful control of the disease is exemplified by elimination of LF in China [38,39] and South Korea [40,41]. In China, the programme started in the 1950 s, with a sustained government commitment to the elimination of lymphatic filariasis. Interventions included mass distribution of a combination of diethylcarbamazine (DEC) tablets and DEC-fortified salt accompanied by intensive surveillance to monitor the prevalence of infection. Building on the successful experience and taking advantage of the available drug albendazole (donated by GlaxoSmithKline for areas coendemic with onchocerciasis), the Global Program to Eliminate Lymphatic Filariasis (GPELF) was established in 1997 to eliminate the disease as a public health problem worldwide by the year 2020. The strategy used is annual MDA with albendazole and DEC or albendazole and ivermectin to over 80% of the entire population at the risk of infection. Since 2000, GPELF has delivered MDA to around 700 million people worldwide, averting 32 million DALYs and an estimated 6.6 million newborns being prevented from the disease [8,42]. Data collected from the sentinel sites in several countries showed that the prevalence of microfilaraemia had been reduced by 60% to 100% after 5-6 rounds of MDA [42], and that Egypt, after 5 rounds of MDA at high coverage, is likely to join the list of countries that has successfully eliminated LF [43]. Despite the progress made, 20 out of 71 endemic countries that require MDA are still yet to start the control programme.

### Schistosomiasis

Schistosomiasis, or bilharzia, caused by the schistosome trematode, is one of the most debilitating helminthic diseases among rural populations. There are three major species that cause tremendous human morbidity, *Schistosoma haematobium *and *Schistosoma mansoni *predominantly in Africa, and *Schistosoma japonicum *in the Far East. Schistosomiasis can cause a wide range of symptoms and consequences depending on the species, the worm burden and the length of time infected [44]. Globally there are over 200 million people infected with the disease with many more at the risk of being infected, and the majority are in sub-Saharan Africa [45].

In China, the national control programme for schistosomiasis has restricted the disease to very limited areas with relatively low prevalence via decades of comprehensive control effort [46,47]. In Brazil and Egypt, similar successes in schistosomiasis control have also been achieved through MDA campaigns [48,49]. The current control strategy recommended by the WHO is to control morbidity due to schistosomiasis by MDA with praziquantel, targeting mainly school-age children and adults at high risk of infection. Preschool children and infants are also being considered for inclusion in the target population [50]. Unlike for other major NTDs there has been limited drug donation and so praziquantel has had to be purchased, although it is now available at low cost. In 2002, the Schistosomiasis Control Initiative was established with support from the Bill & Melinda Gates Foundation, and has since delivered over 40 million treatments in sub-Saharan Africa using praziquantel [51]. The treatment made a significant impact on infection [52,53] and morbidity [54,55] in the targeted populations. Currently there are 13 countries in sub-Saharan Africa that are implementing integrated NTD control programmes in which schistosomiasis control is an important component, however the majority of endemic countries are still yet to start such integrated control programmes.

### Soil-transmitted helminthiasis

Soil-transmitted helminthiasis (STH) is caused by a group of parasitic nematode worms (most importantly, hookworms, *Ascaris lumbricoides *and *Trichuris trichiura*) through ingestion of parasite eggs or contact with skin-penetrating larvae that are present in the contaminated environment. These diseases are by far the most common NTDs in the developing world [3], affecting school-age children in particular and causing anaemia and other forms of malnutrition [18-22,56]. The control strategy is morbidity control through MDA using albendazole or mebendazole. Generally, control of STH is integrated into other programmes such as nutrition or school health as a deworming component, and is often part of schistosomiasis control programmes or as a collateral benefit from LF control programmes, as the same drug is used. Addition of mebendazole to vitamin A supplementation expanded the treatment to children of 12-59 months with a high coverage [57]. Children Without Worms (CWW) was established as a partnership between Johnson & Johnson and the Task Force for Global Health to leverage the donation of mebendazole from Johnson & Johnson to advocate for comprehensive and sustainable control of STH with a focus exclusively on school-age children http://www.childrenwithoutworms.org. Despite these efforts, less than 20% of the at-risk school-age population receive deworming treatment worldwide [58]; this is far short of the World Health Assembly's target to regularly treat 75% of school-age children at risk of morbidity due to schistosomiasis and STH infections by 2010 [59]. As for schistosomiasis control, only a few countries are currently implementing or about to implement national integrated NTD control programmes that include STH control.

### Trachoma

Trachoma, a recurrent eye infection caused by the bacteria, *Chlamydia trachomatis*, is the leading infectious (and thus preventable) cause of blindness worldwide. Repeated infection of the conjunctiva lining the underneath of the eyelid leads to scarring and the eventual turning-in of the eyelid, causing the eyelashes to rub against the eye itself. This painful condition, called trichiasis, eventually abraded the cornea and results in irreversible blindness.

Trachoma is endemic in 57 countries, particularly in sub-Saharan Africa, the Middle East and Asia, with the highest prevalence rates reported in sub-Saharan Africa [60]. The disease affects the poorest populations in the world. It is estimated that there are currently over 40 million people with the infectious form of the disease, and 8.2 million people that have trichiasis and are thus at risk of blindness. It is further estimated that over 7 million people have been blinded by trachoma [60].

The WHO, through the Global Alliance for the Elimination of Blinding Trachoma (GET2020), endorses the SAFE strategy (for 'Surgery for trichiasis, Antibiotics to treat infections, Facial cleaning and Environmental improvement to reduce transmission') [61,62] to eliminate this disease as a cause of blindness by the year 2020 [63]. In 1998, the International Trachoma Initiative (ITI), now part of the Task Force for Global Health, was established by the Edna McConnell Clark Foundation and Pfizer, Inc. [64], and has been working with governmental and non-governmental partners to support the implementation of the SAFE strategy in 18 endemic countries through the donation of the drug Zithromax (azithromycin) by Pfizer as the 'A' component of the SAFE strategy. As a result of this donation programme and the implementation of the other components of the SAFE strategy, in 2006 Morocco announced the elimination of trachoma as a public health problem. Several countries such as Ghana, Iran, Oman, Mexico and Saudi Arabia have since followed suit.

Trachoma control is currently included in the integrated control of NTDs but primarily for antibiotic distribution (the 'A' component). Funds to support the other components in the SAFE strategy, particularly surgery for trichiasis, have to be sourced elsewhere.

### Recommendations for a sustainable success in the control of NTDs

#### Long-term commitment

The increasing commitment by the governmental and non-governmental donors in funding the control of NTDs has excited many. In 2006, the US Congress earmarked $100 million for the United States Agency for International Development to target the aforementioned 'tool-ready' NTDs. In 2008, President Bush launched the NTD Initiative with $350 million over 5 years to provide treatment to over 300 million people in developing countries. In 2009, President Obama announced a new global health initiative with $63 billion over 6 years to address a range of global health problems including NTDs. The US Government has since steadily increased funding in NTD control over the last few years, from $15 million per year during 2006-2008, to $25 million in 2009, and to $65 million for 2010. The UK government also announced a commitment of £50 million over 5 years towards NTD control and elimination. In 2009, a $34 million grant was also provided by the Bill & Melinda Gates Foundation to establish regional strategies and funding mechanisms and leverage new investments to control or eliminate NTDs by 2020. Indeed, with the funding provided, many countries have now been able to implement their integrated NTD control programmes.

However, caution must be taken to avoid being overoptimistic and sending the wrong messages to donors for achieving the stated goals over a short period of time. Overoptimism can artificially raise donor expectations, which may not be met during the funding period. Experience shows that a successful control programme needs a long-term effort. It has been decades since schistosomiasis control programmes started in China in the 1950 s with a comprehensive control strategy [47] and in Brazil in the mid 1970 s with (mainly) chemotherapy [48], yet effort in control is still required and ongoing. There have been resurgences (or re-emergences) of schistosomiasis in previously controlled areas [47,65]. With onchocerciasis, it took 15-17 years of biannual MDA plus vector control to reduce the prevalence to a very low level as in Senegal and Mali [35]. It is therefore important that donors are aware that the overall objectives in controlling or eliminating NTDs may not be achieved after one or two rounds of a 5-year funding cycle using the current strategy, and that long-term commitment and investment are needed.

A long-term commitment is also required from the endemic countries. Current integrated control programmes are almost exclusively funded by external sources. It is recognised that the governments of endemic countries have to balance resource allocation for NTD control against a high burden of more dramatic diseases, and as such they rarely allocate budgets for implementation activities of NTD control programmes. However, in-kind contributions have been made as well as the commitment to integrate NTD activities into health systems. For long-term sustainable control, the endemic countries need to progressively take on the responsibility of such control programmes. This will also ensure that the residual levels of NTDs will be manageable within the health system when they cease to be of public health significance following intensive large-scale, relatively expensive, and community based interventions. It is also important for the beneficiary communities to realise that a long-term commitment is needed from them.

#### Partnerships

Successful control of NTDs needs a strong partnership involving all stakeholders across the various sectors. The successes of OCP, APOC, GPELF and GET2020 are all stories of partnerships of donors, the World Health Organization, pharmaceutical industries, governments of endemic countries, communities, and non-governmental developmental organisations (NGDOs). Commitment from the pharmaceutical industries to continue to donate the necessary drugs is also vital. Currently, Mectizan for onchocerciasis treatment will be donated by Merck for as long as is needed and with as much as is needed [66]. Albendazole for LF will be donated by GlaxoSmithKline for as long as necessary to eliminate LF to meet the current target of elimination by 2020 [67]. Pfizer donates Zithromax for trachoma treatment that is necessary to achieve the goal of the elimination of blinding trachoma by 2020. Johnson&Johnson also donates mebendazole for STH treatment. However, the funds provided by donors to deliver these drugs to the populations in need are still not enough [68]. Therefore, in order to better use the limited resources, all the stakeholders within a country including donors and NGDOs, coordinated by governments of endemic countries, need to work together to identify those priority areas most in need and to plan and implement the control activities in a coordinated and harmonised way to avoid repetition and overlapping in both funding and effort. In this partnership, NGDOs have a very important role in assisting countries to raise funds and plan and implement the programmes, as has been demonstrated in onchocerciasis control [69].

#### Concerted action

Successful control of these diseases and the alleviation of the suffering of millions of people from long-term illness, disfigurement, social stigma and marginalisation, and decreased productivity require concerted action.

Concerted action is needed from donors. Currently, the US government, the UK government, the Bill&Melinda Gates Foundation and some other private donors have provided some of the much-needed funds. Although G8 leaders put NTDs on the global health agenda and called for sustained action against NTDs, so far no major funding has come through from other major donor countries. With the improvement of the global economic situation, more donors need to fulfil their commitments to provide additional funds for the cause of NTD control so that efforts can be scaled up to reach all those people in need [70].

Concerted action is also needed from the endemic countries. Many of the NTDs spread across borders. Cross-border transmission of the NTDs is common because of the movement of populations. Expanding control efforts into bordering countries will be critical, as successful NTD control programmes in one country may be undermined by cross-border traffic from neighbouring countries where there are no NTD control programmes [68]. The neighbouring countries can also work together to exchange experience in planning, implementation, training and advocacy [51,71].

#### Comprehensive control measures

The current strategy for integrated control of NTDs is preventive chemotherapy using the available drugs [30] for one group of NTDs that share MDA as a common strategy. Therefore, the current major funding for the integrated NTD control programmes focuses almost exclusively on integrated MDA, ignoring a number of other components that are critical to the control, mitigation and in some cases the elimination of these diseases. For a long-term and sustainable impact, other measures (for example, clean water supply, hygiene and sanitation, behavioural changes, and vector management) must be addressed. The importance of socioeconomic development in NTD control needs to be recognised and appropriate collaboration sought when implementing NTD interventions. While these other measures will need concerted action from all relevant sectors and may not be fully addressed within an individual programme, for the purpose of morbidity control, extreme cases (for example, trichiasis, hydrocele, and elephantiasis) must be cared for and be included in the current funding. The control programme *per se *can actually benefit from such inclusion of case management as it has been shown that including lymphoedema management in the control programme can increase the community compliance with MDA [72]. In the case of trachoma control, the objective is global elimination of trachoma as a blinding disease by year 2020 using the SAFE strategy. In the current major funding, only the 'A' component is supported, that is, MDA with Zithromax, while the 'S', 'F' and 'E' components are overlooked. Without addressing the 'F' and 'E' components, the disease is guaranteed to return in the long term. There are currently 8.2 million people worldwide with trichiasis, the stage of trachoma that leads to irreversible blindness [60]. If funding for surgery for these cases (the 'S' component) is not provided in the major funding, the main objective of GET2020 will never be achieved. There is a similar case for LF elimination for which disability management and prevention is of equal importance in the programme in order to attain the elimination goal. Another disease for which other complementary measures should be included is schistosomiasis. MDA with praziquantel alone showed excellent results in reducing prevalence and intensity of infection in the integrated control programmes [52,53,73], but it also showed difficulties in reducing infection in heavy endemic areas with close proximity to major water bodies [52], as the once a year treatment does not prevent reinfection. Even in areas where prevalence is reduced to very low levels by consecutive MDA, there is still a risk of recrudescence of the disease as shown in China [47,65]. The helminth control programme on Zanzibar Island is another good example of why comprehensive control measures are needed [74,75]. Therefore, for long-term sustainable control, there should be other measures including clean water supply, hygiene and sanitation such as latrine building and use, health education for behavioural change, and snail management if possible.

#### Implementation through the primary healthcare system and capacity building

In most developing countries, particularly in the poorest areas, there is a lack of fully functional infrastructure in the health service system. If strengthened, this system can be comprehensive, flexible in adjusting to changing disease patterns, permanent, and embedded in community life [76]. As an integrated NTD control programme is implemented in a country, it is important to avoid creating a separate parallel health service system relative to the existing one, and to avoid undermining the already under-resourced healthcare systems [77]. In areas with poor infrastructure and poor resources, a programme-oriented approach has been effective to achieve maximum effect in a short period of time in onchocerciasis control using community-directed interventions in APOC. However, for a long-term benefit and sustainable control programme on NTDs, it is vital to integrate NTD control into the existing primary healthcare system as routine health service operations where possible and thus to strengthen the infrastructure of the primary health system through such control programmes [76,78,79]. This is exemplified by the successful integration of NTD drug delivery through the national Child Health Days in Uganda [80,81]. Experience from the national integrated NTD control programme in Sierra Leone showed that implementing the programme involving the existing health service system actually helped rebuild and strengthen the system (M Hodges, personal communication).

The national NTD control programme belongs to the country and the beneficiary communities that need to take the ownership and responsibility over the implementation of the programme. For a successful control programme, one of the most important issues is the in-country capacity to implement such integrated control programmes. Under the current funding structure, an international organisation or an NGDO (or a consortium) is required to provide technical assistance and support in financial management to the national programme. It is vital that the government and the communities, rather than NGDOs, take the lead in planning and implementation of the programme. With the assistance from NGDOs, the programme can start from a limited scale to gain experience and build capacity through training and implementation, and then expand progressively.

## Conclusions

Control of the NTDs has gained momentum in the last few years. The burden of these diseases on over 2 billion people in the poorest areas and the importance of controlling these NTDs for a wide range of benefits are recognised by the international community [70]. While impressive progress has been made in investment and control of the diseases, a long-term commitment from existing donors must be emphasised and funding from more governmental and non-governmental donors must be mobilised in order to ensure that the billions of people afflicted by the NTDs benefit from such a large-scale international effort. With increased funding in the control of NTDs, the NTD control community must remain objective and maintain focus on maximum achievement with currently available resources. The current level of funding for NTD control has never been seen before, but it is still not enough to scale up to all people in all endemic countries, and the duration of assured funding is not known. The stakeholders must work in a coordinated and harmonised way to identify the priority areas and the best delivery approach to use the current funds to maximum effect. Case management and other necessary measures should be supported in the current funding stream in order to achieve the objectives of these disease controls. For a long-term and sustainable effort, control of NTDs should also be integrated into the primary healthcare system and strengthen the needed supporting structures where required.

## Competing interests

The authors declare that they have no competing interests.

## Authors' contributions

YZ drafted the manuscript, and all authors contributed to and revised the manuscript.

## Pre-publication history

The pre-publication history for this paper can be accessed here:

http://www.biomedcentral.com/1741-7015/8/67/prepub
